# Supplementary material for: A deep learning–based algorithm for tall cell detection in papillary thyroid carcinoma
Source: PLoS One. 2022 Aug 9;17(8):e0272696. doi: 10.1371/journal.pone.0272696 (PMC9362950; doi:10.1371/journal.pone.0272696)
Supplement: S1 File — (DOCX) [file pone.0272696.s001.docx]

|  | | Algorithm TC percentage assessment | | | | | | | | | | |  |
| --- | --- | --- | --- | --- | --- | --- | --- | --- | --- | --- | --- | --- | --- |
|  |  | *0*–*9%* | | *10*–*19%* | *20*–*29%* | *30*–*39%* | *40*–*49%* | *50*–*59%* | *60*–*69%* | *70*–*79%* | *80*–*89%* | *90*–*100%* | *Total* |
| Visual TC percentage assessment | *0*–*9%* | *6* | | *23* | *4* | *5* | *2* |  |  |  |  |  | *40* |
|  | *10*–*19%* | *1* | | *1* | *3* | *3* |  |  |  |  |  |  | *8* |
|  | *20*–*29%* |  | | *1* | *2* | *4* | *1* |  |  |  |  |  | *8* |
|  | *30*–*39%* | *1* | | *1* | *5* | *6* | *3* |  |  |  |  |  | *16* |
|  | *40*–*49%* |  | |  |  |  | *1* |  |  |  |  |  | *1* |
|  | *50*–*59%* |  | |  |  | *5* | *1* |  |  |  |  |  | *6* |
|  | *60*–*69%* |  | |  | *1* | *1* | *2* | *1* |  |  |  |  | *5* |
|  | *70*–*79%* |  | |  |  | *1* | *3* |  |  |  |  |  | *4* |
|  | *80*–*89%* |  | |  |  |  | *2* |  |  |  |  |  | *2* |
|  | *90*–*100%* |  | |  |  |  |  |  |  |  |  |  | *0* |
|  | *Total* | *8* | *26* | | *15* | *25* | *15* | *1* | *0* | *0* | *0* | *0* | *90* |

*Supplementary table 1. Interrater agreement between the visually assessed tall cell (TC) percentage score and the algorithm’s TC score of the primary tumors. An interrater agreement analysis was performed and yielded a weighted kappa value of 0.36 (SD=0.058).*

|  | | Algorithm TC percentage assessment | | | | | | | | | | |  |
| --- | --- | --- | --- | --- | --- | --- | --- | --- | --- | --- | --- | --- | --- |
|  |  | *0*–*9%* | | *10*–*19%* | *20*–*29%* | *30*–*39%* | *40*–*49%* | *50*–*59%* | *60*–*69%* | *70*–*79%* | *80*–*89%* | *90*–*100%* | *Total* |
| Visual TC percentage assessment | *0*–*9%* | *2* | | *13* | *10* | *5* |  | *1* |  |  |  |  | *31* |
|  | *10*–*19%* |  | | *1* | *3* | *3* |  |  |  |  |  |  | *7* |
|  | *20*–*29%* |  | | *1* | *2* | *4* | *1* | *1* |  |  |  |  | *9* |
|  | *30*–*39%* |  | |  | *3* | *2* | *2* |  |  |  |  |  | *7* |
|  | *40*–*49%* |  | |  | *3* |  |  | *1* |  |  |  |  | *4* |
|  | *50*–*59%* |  | |  |  | *2* | *2* | *1* |  |  |  |  | *5* |
|  | *60*–*69%* |  | |  | *1* | *1* | *1* |  |  |  |  |  | *3* |
|  | *70*–*79%* |  | |  |  | *2* | *3* |  |  |  |  |  | *5* |
|  | *80*–*89%* |  | |  |  |  |  |  |  |  |  |  | *0* |
|  | *90*–*100%* |  | |  |  |  |  |  |  |  |  |  | *0* |
|  | *Total* | *2* | *15* | | *22* | *19* | *9* | *4* | *0* | *0* | *0* | *0* | *71* |

*Supplementary table 2. Interrater agreement between the visually assessed tall cell (TC) percentage score and the algorithm’s TC score of the relapses. An interrater agreement analysis was performed and yielded a weighted kappa value of 0.25 (SD=0.056).*
