# Supplementary material for: A deep learning–based algorithm for tall cell detection in papillary thyroid carcinoma
Source: PLoS One. 2022 Aug 9;17(8):e0272696. doi: 10.1371/journal.pone.0272696 (PMC9362950; doi:10.1371/journal.pone.0272696)
Supplement: Attachment — Submitted filename: Rebuttal_letter_final.docx [file pone.0272696.s002.docx]

Rebuttal letter

**Reviewer #1:**

**General Comments:**

**The authors aimed to train a deep learning algorithm to detect the proportion of tall cell variants in papillary thyroid carcinomas. Comparisons were made to scoring by a human investigator, 71 samples were used for training and testing, with an additional 90 samples used for validation.**

*The primary aim of the study was to train a deep learning-based algorithm to detect tall cells in papillary thyroid carcinoma (PTC) and compare the algorithm results to that of a human investigator as well as the outcome of the patients. A total of 100 whole-slide images (WSI) from 100 separate PTC patients were used for training and testing the algorithm; 70 patients and corresponding WSIs from the Hospital District of Helsinki and Uusimaa and the remaining 30 patients and WSIs from the Cancer Genome Atlas Program (TCGA). The validation series consisting of 90 PTC WSIs from 90 separate age-, sex, and T-stage matched PTC patients was used to validate the trained algorithm and how it performed compared to a human investigator.*

*Furthermore 71 tissue slides from 71 separate relapses from the 34 patients with an aggressive disease (figure 1) in the validation series were analysed. As a secondary aim of the study, these tissue slides representing cases with relapse were also assessed by the algorithm as well as the human investigator to study whether the TC morphology of the primary tumor could also be seen in the relapse morphology.*

Figure 1. CONSORT flow diagram of the datasets included in the study.

**Specific Comments:**

**1. Methods, Patient Series, Training Series: The algorithm is reported to have been trained on 100 whole slide images (70 from HUH; 30 from TCGA), however it is not explicitly stated whether these are from separate individuals. Please indicate if these are from separate subjects and, if not, how many unique individuals provided samples.**

Rebuttal: *The 100 total samples in the training set all originated from separate patients.*

*All formalin-fixed paraffin embedded (FFPA) tissue samples from all 70 patients originating from the Hospital District of Helsinki and Uusimaa (HUS) were collected and reviewed by two researchers (S.S., J.A.). One representative FFPA tissue block was selected for each of the patients. New tissue slides were cut, stained with hematoxylin and eosin (HE) and further digitally scanned and imported to the image management platform used to train the algorithm (Aiforia, Aiforia Technologies Oy, Helsinki, Finland). In order to improve the generalizability of the trained algorithm, 30 additional tissue slides originating from the Cancer Genome Atlas Program (TCGA). These 30 slides were from cases with separate patients and were chosen based on visual assessment. Please see the CONSORT diagram of the different datasets used in the study (figure 1).*

Adjustments made: Manuscript edited, and figure added to clarify that the training samples originated from separate patients (page 3, Methods section, 2.1.1. Training series, row 2 and the newly added figure (figure 1) CONSORT flow diagram on page 5, Methods section, 2.3. Training of the deep learning algorithm, rows 3-5, figure 1, page 4).

**2. Methods, Patient Series, Testing Series: The authors include a separate validation set of 90 patients and were careful to include both cases (with adverse outcomes) and controls. This is sound practice. However, the authors mention that 71 tissue samples were obtained only from those in the adverse outcome group. It is not clear whether the validation sample size is 90 or 71, and if the latter, what the point of including the controls was.**

Rebuttal: *The validation set included 90 patients; 34 patients with an adverse outcome and 56 age-, gender-, and T stage matched control cases.*

*The main objective of the study was to validate the trained algorithm using a case-control approach and to study how the TC score of the algorithm correlated with disease outcome. A secondary objective of the study was to test a hypothesis that the tall cell composition is increased in relapse samples compared to the corresponding primary tumor sample. In order for us to analyse this, we collected all relapse/metastasis FFPA tumor samples (e.g. local tumor recurrence or lymph node metastases) from the 34 patients with an adverse outcome. In the same manner as with the primary tumor samples, all FFPA samples were reviewed by two researchers (S.S., J.A.) and one representative FFPA tissue block was selected for each of the 71 relapses. The FFPA were then cut and the tissue samples were stained with HE digitized and imported to an image management platform. After the algorithm was validated on the 90 primary tumor samples in the validation set, the algorithm then analysed the 71 relapse tumor samples. In the study, we concluded that the tall cell morphology is retained but not increased in relapse samples compared to the primary tumor morphology.*

Adjustments made: Text edited to outline the aims of the study more clearly (Page 3, Introduction section, rows 22-24). A new subheader “Relapse series” was inserted to highlight that these WSIs are indeed not included in the validation series (page 5, Methods section, 2.1.3. Relapse series). Also, the “digitization of slides” subheader was deleted and embedded into the “Validation series” text (Page 4, Methods section, 2.1.2 Validation series, rows 7-16). A consort diagram added to illustrate the different datasets more clearly (figure 1).

**3. Methods, Training of Deep Learning Algorithm: The authors mention 2,674 manual annotations were used for training and 296 were used for testing. The large numbers involved help justify the somewhat extreme 90/10 training/testing set ratio (something like 70/30 is more standard), but it is not clear where these large numbers come from, given that the previously reported sample size were 90.**

Rebuttal: *In total, the training set consisted of 100 tumor samples; 70 samples originating from Helsinki University Hospital and the remaining 30 from the TCGA database. However, the algorithm was trained on 2,674 manual annotations of regions of interest within these 100 patient samples. The manual annotations were done by one researcher (S.S.).*

*The reviewer brings up a good point that a training/testing set ratio or 80/20 or even 70/30 is more common and perhaps more standard. However, in a 90/10 split was justified based on the quantity of manual annotations in the training set, were as many as 2,674 and that the test set consisted of 296 manual annotations of regions of interest. A power calculation is reported below to illustrate that for analysis of the sensitivity of the algorithm on the annotation level, this number can be considered sufficient.*

Changes made: Added a new figure of a CONSORT flow diagram outlining the datasets used in the study (figure 1, page 4).

**4. Methods, Training of Deep Learning Algorithm: The authors performed their training/testing split once and missed an opportunity to determine the stability of their algorithm and the validity of their results by performing different splits of the data into training/testing and reproducing the model. Please justify why only a single split was made.**

Rebuttal: *If we assume the prevalence of TCs to be 30% (estimated based on visual assessment) in the manual annotations used for training and testing, we would need 244 manual annotations in the test set to reach the acceptable +/-5% width of the 95% confidence level of the estimated sensitivity if the expected sensitivity is 95% according to our calculations. Thus, the 296 manual annotations exceed this number and we therefore deemed that one split sufficed. Given the sensitivity level for detection of TC in the test set, we expect a similar sensitivity level i.e. 95% in the validation set. However, the reviewer is correct in that multiple splits could perhaps have determined the stability of the model in more detail.*

**5. Methods, Statistical Analysis: The methods appear sound, though they are slightly under-reported. For instance, the log-rank test and proportional hazard regression model are used for survival analysis, but for what purpose? Please specify the outcomes and fixed effects, as well as the goals for these analyses. The reporting for exact tests and kappa statistics in the same paragraph are excellent examples of the detail needed.**

Rebuttal: *As death from disease is a rather rare event in PTC, relapse was used as the primary outcome in survival analysis of the data. An event in relapse-free survival was defined as time from diagnosis to relapse (Increase in serum thyroglobulin levels or histological confirmation). Patients who died of other causes or otherwise were censored (e.g. treatment moved to another hospital district).*

Changes made: Clarification of parameters used in survival analysis added (page 7, Methods section, 2.4. Statistical analysis, rows 9-12)

**6. Methods, Statistical Analysis: There is shockingly little detail provided about the neural network / deep learning approach. Input and output nodes, the number of hidden layers, the number of neurons, are not reported or discussed. Please provide these details – needed for reproducibility – along with justification for those choices. A Figure would also be helpful.**

Rebuttal: *The exact architecture of the artificial neural network is not available and remains proprietary to the provider of the software (Aiforia Technologies Oy, Helsinki, Finland). This could be considered a limitation and has been added as such in the manuscript. However, as the platform is commercially available, it is possible for other researchers to access and use the platform for training of deep learning-based algorithms. Also, the image augmentation details are provided in the manuscript which makes the method fully reproducible.*

**7. Results: In general, the findings are thoroughly and adequately reported. It is not precisely clear what role the validation sample played in generating them. Please state which results are from testing and which are from validation.**

Rebuttal: *The validation set in the study was used for survival statistics and to compare how the algorithm’s scores compared to that of a human investigator. The validation set was a held-out set and thus not included in training or testing of the algorithm.*

Changes made: Added figure 1, page 4, and edited the text according to earlier comments to clarify this.

**8. Results: The authors do not appear to have calculated sensitivity and specificity for the validation set. This seems an important omission. Please report or state how and why the data preclude such calculations.**

Rebuttal: *The sensitivity and specificity was calculated based on the manual annotations in the test set. The validation set had no ground truth TC labels, and a sensitivity and specificity could therefore not be calculated in the validation dataset.*

*Currently, the TC score is assessed visually by the pathologists mostly using traditional microscopes. The assessment is done visually on slide level, as it would be near impossible for a human to assess the TC score on cell level throughout the whole slide. The TC score is then usually reported with 10% increments. The human TC scoring in the study was performed in the same way; the representative slides were evaluated and a TC score was reported for each representative slide. The algorithm in the study consisted of two layers; first, tumor tissue was detected and secondly both TC area and non-TC area were registered within the tumor tissue area. The second layer of the algorithm analyzed the tissue on a much higher magnification resulting in cell level analysis. That is why, in the validation set, no ground truth annotations existed and therefore no specificity nor sensitivity could be calculated.*

**9. Discussion: The authors state several strengths of their work without explicitly stating any limitations. Surely there must be some!**

Rebuttal: *The validation series of the study were all collected from within the same hospital district. During the time of sample collection, the hospital district offered a rather aggressive initial treatment protocol to adequately treat all aggressive cases but maybe overtreated the more indolent PTCs. This, in turn, could lead to misclassifying of the cases included in the study. As mentioned in the discussion section, we highly recommend multicenter studies in the future to limit the impact of treatment protocols within a specific hospital districts.*

Adjustments made: Explicitly mentioning the limitation of the study cohort (Page 12, discussion section, rows 7-8)

**Reviewer #2:**

**Interesting premise, but I have the following questions about study design:**

**In the validation set all samples were from patients with at least 5 years follow up. This means that any patients with a cancer with a death or other event that lead to loss of follow up by 5 years were not included, thus the population was a healthier population that the expected population with this cancer. How was this bias addressed in the study?**

Rebuttal: *The patients in the validation set included patients that were diagnosed with papillary thyroid carcinoma (PTC) between 2003-2013. By design, the follow-up time extended to 2018, which allowed for all patients included in the study to have a 5-year follow-up time. Patients who died of other causes than PTC or for other causes were censored within 5 years from diagnosis date were also included in the study. This is how, even though we allowed for a minimum 5-year follow-up, some patients had a shorter follow-up and the range of follow-up for the study was 2.1 years to 15.8 years (median 10.1 years) which is stated in the manuscript (page 5, Methods, Validation series, rows 6-7).*

Adjustments made: Text edited to clarify the follow-up time (page 4, Methods section, 2.1.2. Validation series, rows 2-3)

**Patients with an adverse outcome - what was the time frame for this observation?**

Rebuttal: *In the present study, an adverse outcome was defined as PTC cases having at least two recurrences (histological confirmation or serum thyroglobulin elevation during follow-up), having distant metastases or patients who died from PTC. Distant metastases at primary diagnosis was classified as an adverse outcome disease. A PTC was also defined as having an adverse outcome if the PTC distally metastasized, recurred more than twice or the patient died of the disease during follow-up.*

**How was the matching accounted for in the analyses?**

Rebuttal: *Both the human investigator and the deep learning-based algorithm were blinded to the clinical outcome and thus also to the matching when giving TC scores to the cases.*

**How was median relapse free survival assessed? What events were included/ censoring events?**

Rebuttal: *Relapse free survival was defined as time from diagnosis to first relapse. A relapse was defined as serum thyroglobulin elevation during follow-up or histological confirmation. Patients for whom follow-up ended before relapse for different reasons (e.g. moving to another hospital district or death from other disease) were censored in the survival analysis.*

Adjustments made: Clarification of text on page 7, Methods section, 2.4. Statistical analysis, rows 9-12)

**Table 1: include percentages to show if the rates were similar in the two groups for categorical variables.**

Changes made: Percentages added to appropriate variables in table 1.

**Definition of overall survival?**

Rebuttal: *Overall survival was defined as time from diagnosis to death from any cause.*

Adjustments made: Clarification of text on page 7, Methods section, 2.4. Statistical analysis, rows 9-12)
