# Supplementary material for: A deep learning–based algorithm for tall cell detection in papillary thyroid carcinoma
Source: PLoS One. 2022 Aug 9;17(8):e0272696. doi: 10.1371/journal.pone.0272696 (PMC9362950; doi:10.1371/journal.pone.0272696)
Supplement: Attachment — Submitted filename: Revised2_Rebuttal_letter.docx [file pone.0272696.s003.docx]

Rebuttal letter

**Reviewer #1:**

**While the authors responded to my comment (#6, reproduced below), they did not in fact mention the proprietary nature of the neural network approach in the limitations section.**

*Rebuttal: The architecture of the algorithm is not available in exact detail, so it would indeed not be possible to recreate the model without knowing the algorithm structure. With that said, the image management and machine learning platform used in the study is commercially available for anyone to use. As the parameter settings that can be changed in the platform (e.g., feature size, iterations, image augmentations etc.) have been described in detail in the manuscript, the method is considered fully reproducible.*

*Changes made: It is now mentioned that the exact architecture of the algorithm is proprietary and that it could be considered a limitation. (Page 12, Discussion section, rows 26-29)*

**Rebuttal #2:**

**1. "How was the matching accounted for in the analyses?" - this requires a statistical analyses response. For example - In the Cox proportional hazards regression, adjustment was made for the matching variables of age, gender, tumour stage...
Table 3 could be updated to include multiple variable analyses adjusted for age group, gender, tumour stage.**

*Rebuttal: The matching of the cases was done when collecting the samples for the study. Thus, the entire study, including the statistical analyses, were affected and accounted for in the study. In Cox multivariate regression analysis, the model could not be fitted with all four variables the reviewer suggested due to diminishing power and overfitting. We performed a Cox multivariate regression analysis adjusted for age (at 45 years of age threshold) and updated the manuscript and table 3 accordingly.*

*Changes made: Included the recent changes in the manuscript and updated table 3 according to the findings. (Page 9, results section, 3.3. Algorithm-based tall cell score and survival subsection, rows 8-11 and 3.4 Visually assessed tall cell score and survival, rows 10-11, page 12, Discussion section, rows 11-18. Table 3, page 10, reworked and corrected)*

**2. Is it possible that some controls would be cases if observed for a longer period of time? How did 14 patients experience relapse, but were considered controls (if followed for longer, could a second relapse have been observed, making them cases)? It seems that the two groups were linked with the relapse free survival time - thus any analysis of the effect of group on relapse free survival would be biased. It would not be possible to define which group a patient would fall into at baseline.**

*Rebuttal: Papillary thyroid carcinoma (PTC) often has a great prognosis, even with local recurrence. Thus, we designed the present study such that an adverse outcome was defined as two or more relapses (thyroglobulin elevation or histological confirmation), distant metastasis (at primary diagnosis or during follow-up) or death from PTC. Each case with an adverse outcome was matched with one to two control cases matched by age at diagnosis (within 10 years), gender, and tumor stage. The cases were collected between 2003 and 2013 and follow-up was ended 2018. This allowed all included cases at least a 5-year follow-up. By design, this cohort includes a concentrated number of cases with an aggressive disease and as such does not represent the general population. As tall cell variants are rather rare, the concentration of aggressive cases allowed us to study the prognostic impact of tall cells with a rather small sample size which was the aim of the study design.*

*In the survival analysis the cohort was analyzed as a whole and not grouped by aggressive or control. For example, analyzing a 30% tall cell threshold, the only grouping was whether the patient had less or more than 30% tall cells. Thus, the aggressive vs. control grouping would not affect the survival analysis of tall cell thresholds.*

*We hypothesized that patients with a single local recurrence does not have an aggressive disease and that a single recurrence could also be a result of inadequate resection of tumor in the primary surgery. It is possible that some of the patients in the control group could have been diagnosed with additional recurrences if observed for a longer period and longer follow-up periods are thus generally recommended. However, we felt that a 5-year follow-up was adequate for the scope of this study.*

*The cohort was retrospectively collected and most of the criteria for an aggressive disease indeed occurred during follow-up and would have been unknown at time of diagnosis. The reviewer correctly points out that it would not have been possible to define which group a patient would fall into at the time of diagnosis based on the aggressive disease criteria used in the study. However, our aim was to examine tall cell thresholds and how the tall cell scores correlated with outcome. As we concluded in the study, a 30% tall cell threshold did indeed correlate with a reduced relapse free survival. This could have been observed at the time of diagnosis as we studied the same histological tissue blocks that were used in the initial diagnosis. We argue that cases with 30% TC or higher should be treated more aggressively as they correlate with a more aggressive disease.*

*In the future, the algorithm presented here could be used by pathologists in the diagnosis of tall cell variants of papillary thyroid carcinoma and further studied using a prospective cohort.*

*Changes made: Discussion and conclusion sections edited to highlight the findings in the survival analysis (page 12, Discussion section, rows 11-18 and page 13-14, Discussion section, last paragraph of the manuscript)*

**3. In table 1 median age at diagnosis for cases is 43.4 years, but in text above it says 41.0 years - which is correct? Similar 41.7 and 41.5 are both quoted for the controls - which is right?**

*Rebuttal: Both numbers are correct since different numbers are reported; In the text, the median age is reported, while in the table the mean age is reported. This understandably cause confusion and we will edit the manuscript to be more consistent.*

*Changes made: corrected the table so the median age is reported instead of the mean to avoid confusion (page 5, table 1, row 11)* **4. Figure 5: the third line in the number at risk - the label should be >=30% rather than 10% to match with the legend.**

*Rebuttal: The reviewer correctly points out an error in the figure.*

*Changes made: Figure 5 corrected to match the figure legend.*  **5. Include in table 3 the three group split as shown in figure 5, including univariate and multiple variable analyses with this grouping.**

*Changes made: Table 3 changed according to the reviewer’s suggestion. (page 10)* **6. The question is - is a three group split better than a two group split - and this is difficult to tell with these analyses.**

*Rebuttal: The WHO suggests using a 30% TC threshold, thus basically recommending using a two-group split in clinical practice. This is probably preferable in clinical practice as it is very difficult for human observers to adhere to the strict three-group split used in the study (under 10%, 10-29%, and over 30%).*

*The idea of the three-group split in the statistical analysis was to separate the cases with a very small number of TCs (under 10%) which we assumed would have the best prognosis. This seems to be a valid assumption based on figure 5. However, only 8 cases had less than 10% TCs and only one relapse was observed within this group. Having separated out the cases with very few TCs, we could then study the difference between some TCs (10-29%) versus the cases with a lot of TCs (over 30%). Again, based on figure 5, there seem to be a difference in relapse between these two groups as well.*

*The reviewer asks a crucial question and to summarize; Based on our results, when using the algorithm, a 30% TC threshold seem to be the correct threshold to use when diagnosing TCV, which is in line with WHOs current recommendations. However, all cases with TCs (≥10%) should be included in the pathologist’s report as these cases also seem to correlate with a reduction in relapse free survival.*

*Changes made: Conclusion edited to highlight these findings (Page 13-14, Discussion section, last paragraph of the manuscript).*

**7. In the definition of relapse free survival I assume that relapse and death are events, end of follow up is a censoring variable? It is not clear in this sentence.**

*Rebuttal: relapse free survival is the time from diagnosis to relapse as described on page 7, Methods section, 2.4 statistical analysis subsection rows 9-10. The event is thus relapse and censoring events are e.g., end of follow-up or death.*
